# Inhibiting cell migration and cell invasion by silencing the transcription factor ETS-1 in human bladder cancer

**DOI:** 10.18632/oncotarget.7192

**Published:** 2016-02-04

**Authors:** Li Liu, Yuchen Liu, Xintao Zhang, Mingwei Chen, Hanwei Wu, Muqi Lin, Yonghao Zhan, Chengle Zhuang, Junhao Lin, Jianfa Li, Wen Xu, Xing Fu, Qiaoxia Zhang, Xiaojuan Sun, Guoping Zhao, Weiren Huang

**Affiliations:** ^1^ Key Laboratory of Medical Reprogramming Technology, Shenzhen Second People's Hospital, The First Affiliated Hospital of Shenzhen University, Shenzhen, China; ^2^ Urology Department, Qingyuan People's Hospital, The Sixth Affiliated Hospital of Guangzhou Medical University, Qingyuan, China; ^3^ Department of Urology, Peking University First Hospital, Institute of Urology, Peking University, National Urological Cancer Center, Beijing, China; ^4^ Shantou University Medical College, Shantou, China; ^5^ Shanghai-MOST Key Laboratory of Health and Disease Genomics, Chinese National Human Genome Center at Shanghai, Shanghai, China

**Keywords:** bladder cancer, ETS-1, oncogene, cell migration, cell invasion

## Abstract

As one of the members of the ETS gene family, the transcription factor v-ets avian erythroblastosis virus E26 oncogene homolog 1 (ETS-1) plays key role in the regulation of physiological processes in normal cells and tumors. In this study, we aimed to investigate the relationship between the transcription factor ETS-1 and malignant phenotypes of bladder cancer. We demonstrated that ETS-1 was up-regulated in human bladder cancer tissue compared to paired normal bladder tissue. In order to evaluate the functional role of ETS-1 in human bladder cancer, vectors expressing ETS-1 shRNA and ETS-1 protein were constructed *in vitro* and transfected into the human bladder cancer T24 and 5637 cells. Our results showed that the transcription factor ETS-1 could promote cell migration and cell invasion in human bladder cancer, without affecting cell proliferation and apoptosis. In conclusion, ETS-1 plays oncogenic roles through inducing cell migration and invasion in human bladder cancer, and it can be used as a therapeutic target for treating human bladder cancer.

## INTRODUCTION

Human bladder cancer is one of the most common cancers worldwide [1]. Although methods for early diagnosis of bladder cancer and more suitable surgeries were emerged, the mortality rate has not been decreased effectively [2]. One of these important reasons is that the potential mechanisms causing cancer progress are still poorly understood. Therefore, searching novel reliable biomarkers and revealing the molecular mechanism of bladder cancer for developing effective therapy are necessary.

As the first found member of the ETS transcription factors family, the transcription factor v-ets avian erythroblastosis virus E26 oncogene homolog 1 (ETS-1) plays diverse roles in physiological and pathological processes, such as embryonic development, angiogenesis, and hematopoietic differentiation [3, 4]. Furthermore, deregulated ETS-1 also has been observed in the carcinogenesis, invasion, and progression of various tumors [5, 6, 7]. One of the potential mechanisms of ETS-1is to activate the transcription of several proteases, such as matrix metalloproteinases (MMPs) and urokinase-type plasminogen activator [5, 6, 7]. However, the functional character of ETS-1 in human bladder cancer is still unclear. Therefore, we aimed to study the expression pattern of ETS-1 and to determine its functional role in human bladder cancer.

## RESULTS

### ETS-1expression level was increased in bladder cancer

**Figure 1:**
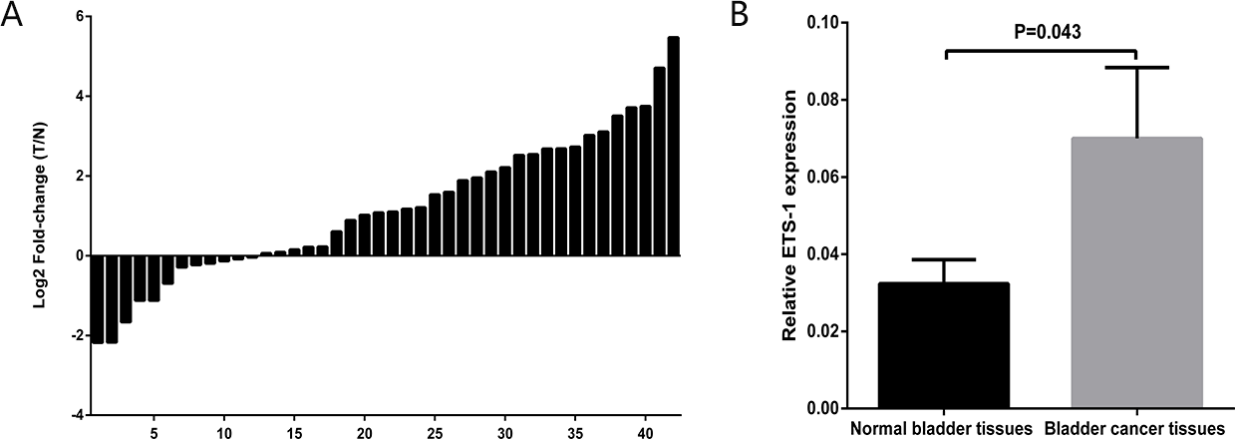
ETS-1expression level was increased in bladder cancer The relative expression levels of ETS-1were detected using Real-Time qPCR in 42 bladder cancer patients. (**A**) The height of the columns in the chart represents the log2-transformed fold changes (bladder cancer tissue/normal bladder tissue) of ETS-1 expression in 42 patients with bladder cancer. (**B**) The expression levels of ETS-1 were significantly up-regulated in bladder cancer tissues compared with matched normal tissues. Data are shown as mean ± SD. *T* represents tumor, *N* represents normal.

The relative expression level of ETS-1 was examined using real-time qPCR in a total of 42 patients with bladder cancer. The ETS-1 expression fold-change (bladder cancer tissue/matched normal tissue) in most patients were greater than zero in Figure 1A. Similarly, as shown in Figure 1B, The expression levels of ETS-1 were significantly up-regulated in bladder cancer tissues compared with matched normal tissues. These results manifested that ETS-1 should play oncogenic role in bladder cancer. After that, we assessed the relationship between ETS-1 expression level and clinical-pathologic characteristics in 42 patients with bladder cancer. In Table 1, the results indicated that ETS-1 expression level was associated with the depth of invasion in these patients with bladder cancer, but it has no relationship with the age, the gender and the grade. These results suggested that ETS-1overexpression may be related to bladder cancer invasion.

**Table 1:** The relationship between ETS-1 level and clinical-pathologic characteristics in 42 patients with bladder cancer

Variable	*n*	ETS-1 level		*P*
	42	low	high	
Age				
< 60	12	4	8	0.957
≥ 60	30	8	22	
Gender				
Male	33	11	22	0.372
Female	9	1	8	
Grade				
Low	11	1	10	0.202
High	31	11	20	
Depth of invasion				
Ta–T1	12	0	12	0.027*
T2–T4	30	12	18	

* *P* < 0.05 was considered significant. Continuous correction chi-square test was used for the statistical analyses.

### Inhibition or activation of the expression of ETS-1 in bladder cancer

**Figure 2:**
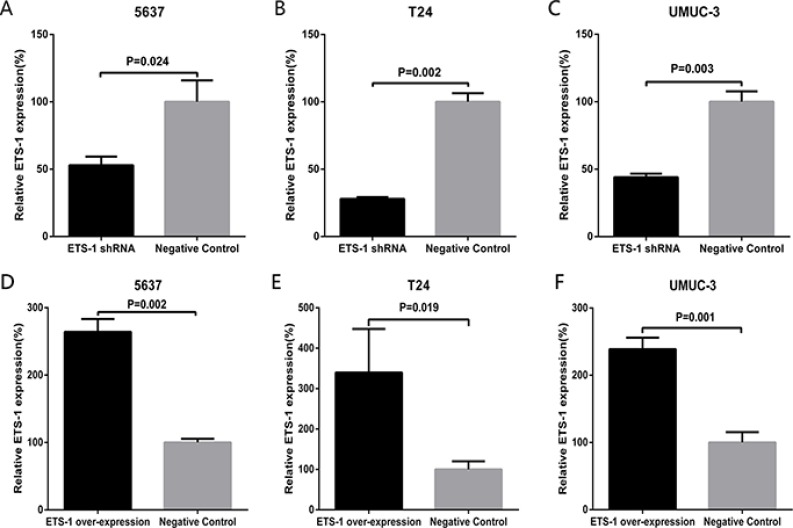
Inhibition or activation of the expression of ETS-1 in bladder cancer The relative expression level was evaluated using real-time qPCR. Compared with negative control group, the ETS-1 specific shRNA obviously down-regulated the expression level of ETS-1 in 5637 cells (**A**) T24 cells (**B**) and UMUC-3 cells (**C**) Compared with negative control group, the ETS-1 over-expression vector obviously up-regulated the expression level of ETS-1 in 5637 cells (**D**) T24 cells (**E**) and UMUC-3 cells (**F**). Data are indicated as mean ± S.D.

Bladder cancer 5637, T24 and UMUC-3 cells were cultured and treated with ETS-1 shRNA or the over-expression vector. The related expression level of ETS-1 was tested by real-time qPCR at 48 hours after treatment. The data indicated that the related expression level of ETS-1in 5637 (Figure 2A), T24 cells (Figure 2B) and UMUC-3 (Figure 2C) was observably down-regulated by the specific ETS-1 shRNA. Also, the over-expression vector increased the expression level of ETS-1in 5637 (Figure 2D), T24 (Figure 2E) and UMUC-3 (Figure 2F) cells.

### ETS-1 promoted bladder cancer cell migration *in vitro*

**Figure 3:**
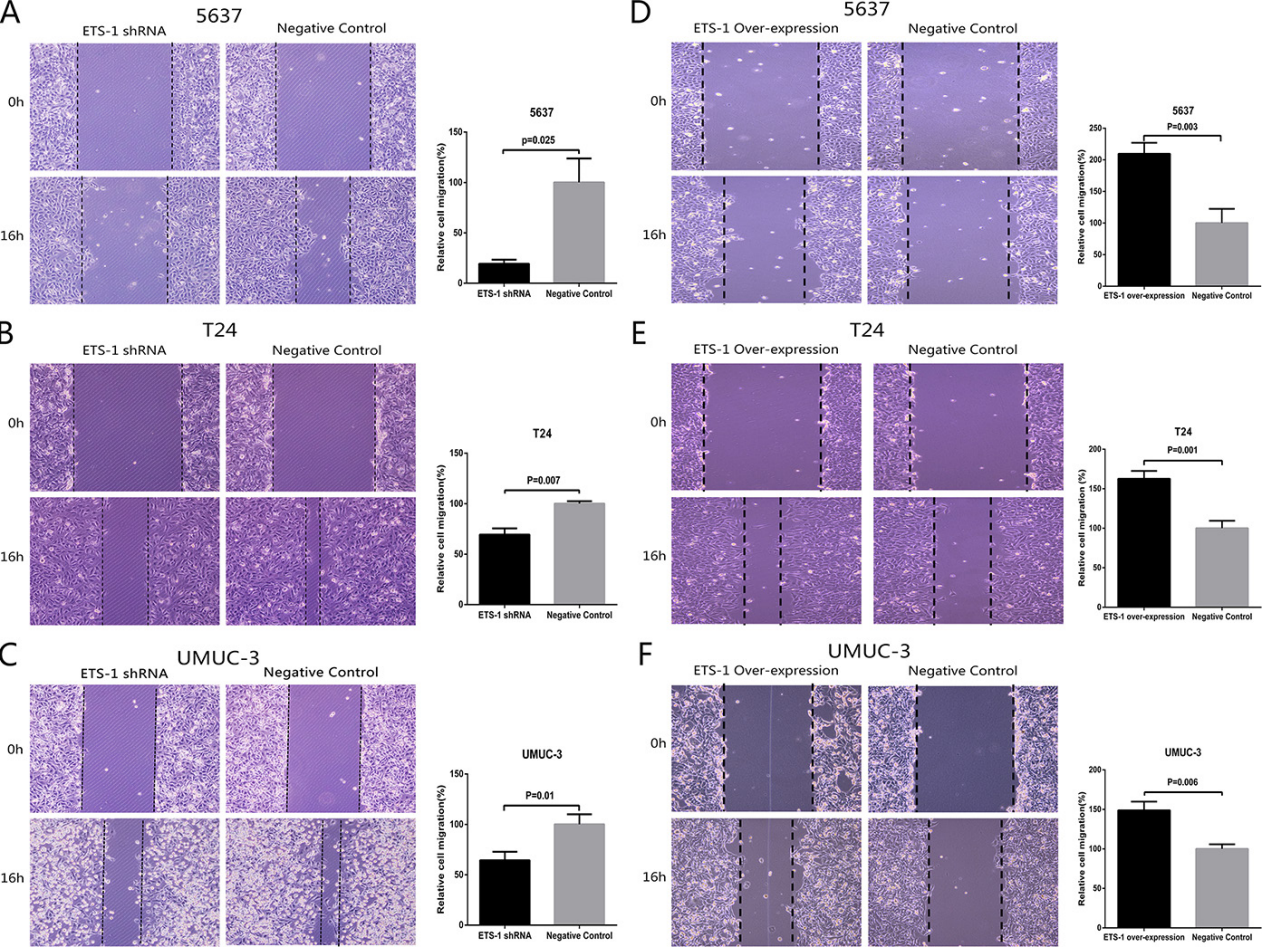
ETS-1 promoted bladder cancer cell migration *in vitro* 5637 (**A**) T24 (**B**) and UMUC-3 cells (**C**) were treated with ETS-1 specific shRNA or negative control shRNA. 5637 (**D**) T24 (**E**) and UMUC-3 cells (**F**) were also transduced with ETS-1 over-expression vector or negative control and cell migration assay was performed as described in Materials and Methods. Representational Figures of each experiment are shown. Data are presented as mean ± SD. Each experiment in both cell lines was performed for three independent times.

In order to evaluate the role of ETS-1 in regulating human bladder cancer migration, bladder cancer 5637, T24 and UMUC-3 cells were treated with ETS-1 shRNA or the over-expression vector and analyzed by the cell migration assay afterwards. Compared to the negative control group, cell migration ability was significantly inhibited by the ETS-1 shRNA in 5637 cells (Figure 3A), T24 cells (Figure 3B) and UMUC-3 cells (Figure 3C). In contrast, cell migration ability was significantly enhanced by the ETS-1over-expression vector in 5637 cells (Figure 3D), T24 cells (Figure 3E) and UMUC-3 cells (Figure 3F). These results demonstrated that ETS-1 positively facilitate the bladder cancer cell migration.

### ETS-1 promoted bladder cancer cell invasion *in vitro*

**Figure 4:**
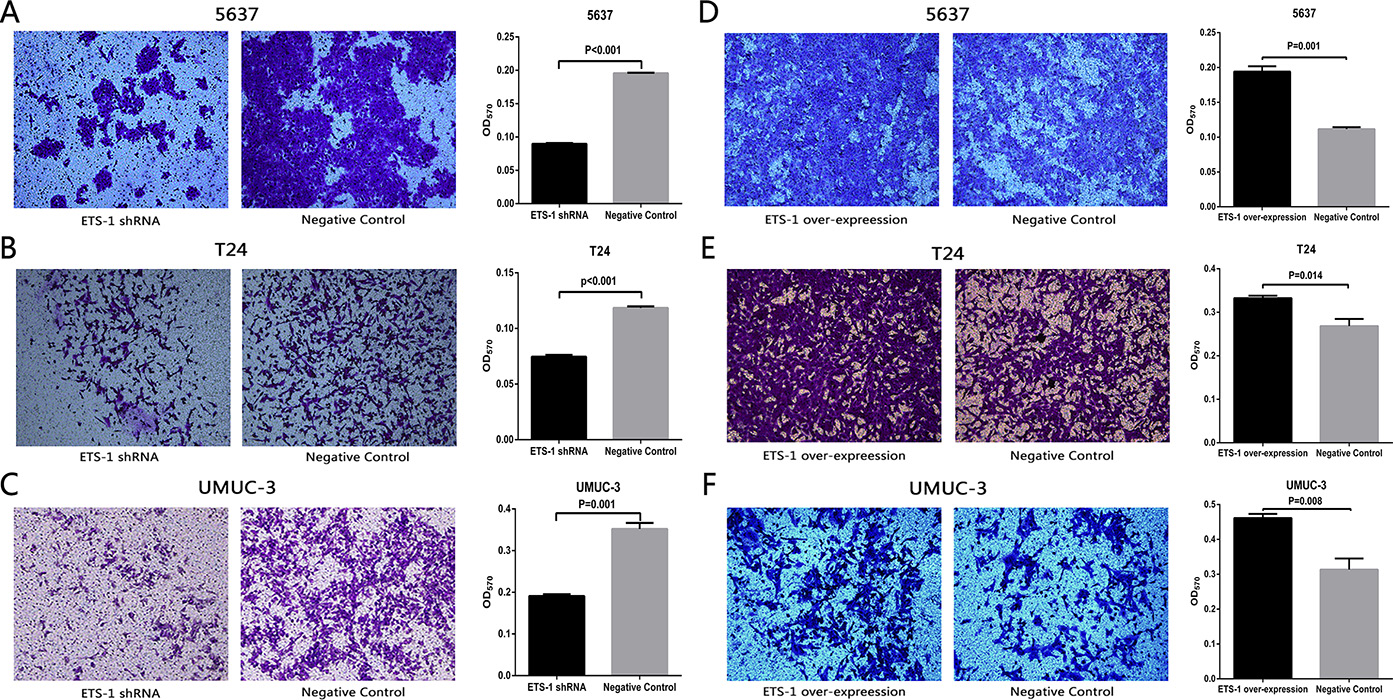
ETS-1 promoted bladder cancer cell invasion *in vitro* Invasion ability of bladder cancer cell was detected by cell invasion assay, whose concrete operating procedures were shown in Materials and Methods. Invaded cells were observed and imaged under the inverted microscope. Afterwards, the invaded cells within each chamber were soaked with 33% acetic acid to wash out the crystal violet, and then the absorbance of crystal violet was measured at the wavelength of 570nm using microplate reader. Cell invasion inhibition was observed in bladder cancer 5637 cells (**A**) T24 cells (**B**) and UMUC-3 cells (**C**) treated with the shRNA. Cell invasion promotion was observed in bladder cancer 5637 cells (**D**) T24 cells (**E**) and UMUC-3 cells (**F**) treated with the over-expression vector. Representational Figures of each test are shown. Data are shown as mean ± SD. Experiments were performed for three independent times.

We further investigated whether ETS-1also promotes cell invasion in bladder cancer. Here, the invasion assay was used to verify our hypothesis. As Shown in Figure 4A–4C, compared with the negative control group, the cell invasion ability was extremely suppressed in 5637 cells, T24 cells and UMUC-3 cells which were transfected with ETS-1 shRNA. As shown in Figure 4D–4F, compared with the negative control group, the cell invasion ability was extremely enhanced in 5637 cells, T24 cells and UMUC-3 cells which were treated with ETS-1 over-expression vector. These data indicated that ETS-1 significantly promote cell invasion in bladder cancer.

### ETS-1 do not control cell proliferation in bladder cancer

**Figure 5:**
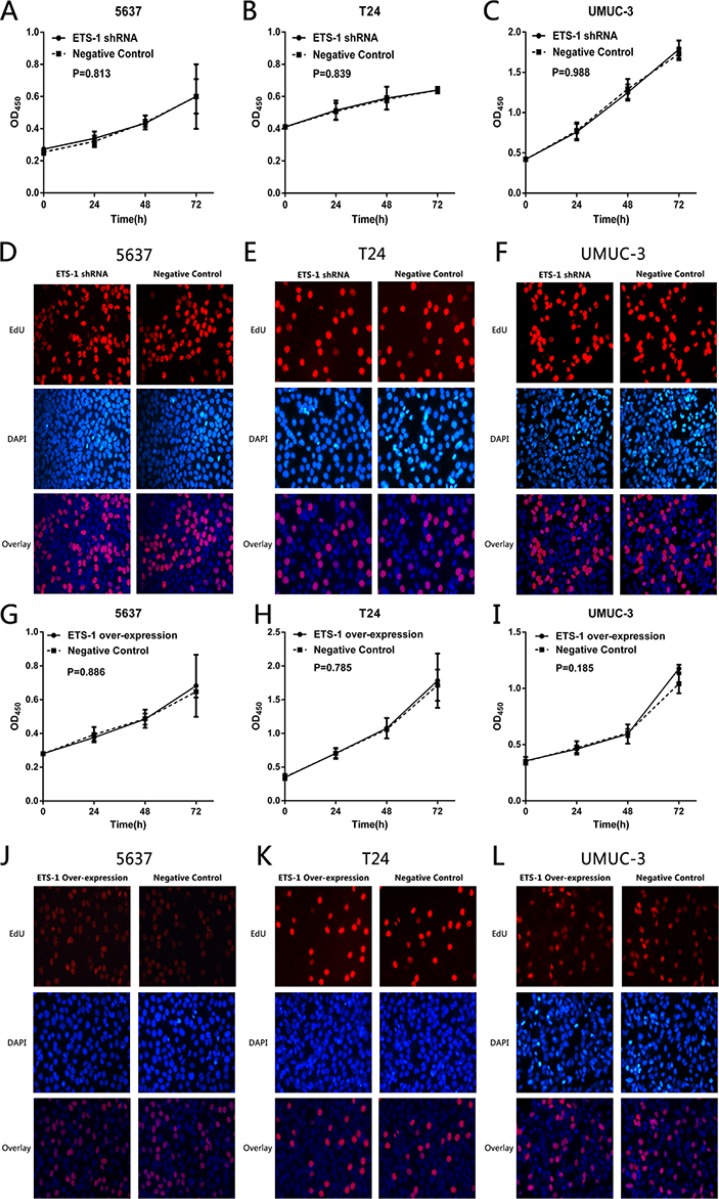
ETS-1 do not control cell proliferation in bladder cancer Cell proliferation was measured by CCK-8 assay and EdU assay. There was no difference in cell proliferation rate between the treated group in bladder cancer 5637 cells (**A, D, G, J**), T24 cells (**B, E, H, K**) and UMUC-3 cells (**C, F, I, L**). The data are presented as mean ± SD. Assays were repeated at least three times.

To explore whether ETS-1is associated with cell proliferation in bladder cancer, the bladder cancer cell lines treated with ETS-1 shRNA or over-expression vector were analyzed by the CCK-8 assay and EdU assay. Both of the two results indicated that there was no difference in the proliferation rate between ETS-1 shRNA or over-expression vector group and negative control group in 5637 cells (Figure 5A, 5D, 5G and 5J), T24 cells (Figure 5B, 5E, 5H and 5K) and UMUC-3 cells (Figure 5C, 5F, 5I and 5L). These results confirmed that ETS-1is not associated with the bladder cancer cell proliferation.

### ETS-1 do not regulate cell apoptosis in bladder cancer

**Figure 6:**
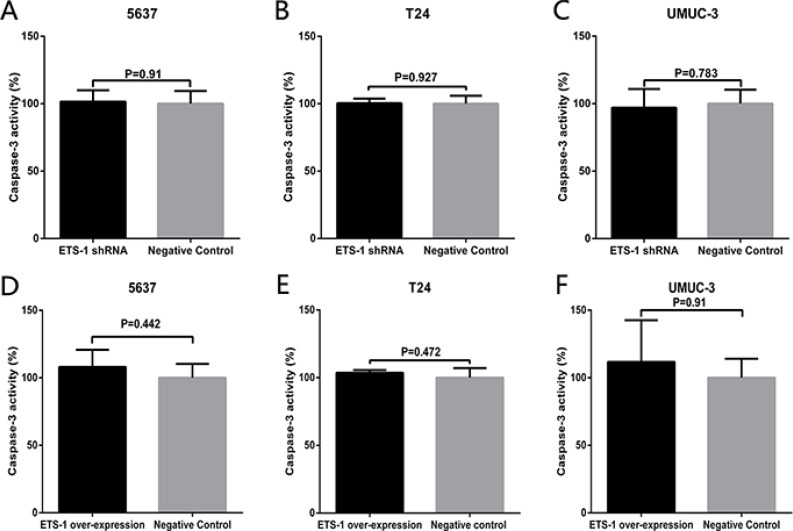
ETS-1 do not regulate cell apoptosis in bladder cancer At 48 hours after treatment of ETS-1 shRNA or over-expression vector, the cell apoptosis changes were determined by ELISA assay. There was no difference in the relative activity of caspase-3 in 5637 (**A, D**), T24 (**B, E**) and UMUC-3 cells(**C, F**). These results are presented as mean ± SD. Tests were repeated at least three times.

Finally, we determined whether ETS-1 inhibit cell apoptosis in bladder cancer. At 48 hours after transfection of ETS-1 shRNA or transduction of the over-expression vector, the relative activity of caspase-3 of the bladder cancer 5637 cells, T24 cells and UMUC-3 cells were detected by thecaspase-3 enzyme-linked immunosorbent assay (ELISA) assay. Regrettably, as shown in Figure 6A–6C, there was also no difference in the relative activity of caspase-3 between ETS-1 shRNA and negative control shRNA transfected group in these three bladder cancer cells. As shown in Figure 6C–6E, there was also no difference in the relative activity of caspase-3 between ETS-1over-expression vector and negative control transduced group in these three bladder cancer cells. The results indicated that ETS-1 has nothing to do with the bladder cancer cell apoptosis.

## DISCUSSION

ETS-1, one of the ETS transcription factor members, is known to play important roles in a number of physiological and pathological processes, such as embryonic development, angiogenesis, and hematopoietic differentiation [3, 4]. For example, ETS-1 is essential for Connective Tissue Growth Factor (CTGF/CCN2) induction mediated by TGF-β1 in Osteoblasts [8] and ETS-1 is significant for EBNA3C-mediated up-regulation of AK-B transcription, which leads to degradation of p53 [9]. Furthermore, ETS-1 is expressed in many tumors and activates many cancer related genes that promote the malignant transformation and progression of tumors, including invasion, metastasis and neoangiogenesis [10–12]. In gastric tumors [13] and oral squamous cell carcinomas [14], the deregulation of ETS-1 was related with the invasive behavior and cancer metastasis. In addition, ETS-1was responsible for the malignant progression and invasion of meningiomas and human astrocytic tumor [15, 16]. Over-expression of ETS-1was also reported in human breast cancer and angiosarcoma of the skin [17, 18].

In our study, we found that the expression level of ETS-1 was significantly up-regulated in bladder cancer tissues compared with matched normal tissues. These results in our research manifested that ETS-1 is likely to play oncogenic role in bladder cancer. To further understand the biological functions of ETS-1 in bladder cancer, the ETS-1 specific shRNA and over-expression vector were designed to treat bladder cancer cells, and then we used scratch assay and invasion assay to detect the cell migration ability and the cell invasiveness. As expected, ETS-1 promoted cell migration and cell invasion in the related bladder cancer cell lines. In addition, cell proliferation and cell apoptosis were tested by the CCK-8, EdU and ELISA assay to verify whether ETS-1 influences the cell proliferation and cell apoptosis in bladder cancer. Unfortunately, there were no differences in both the cell proliferation rate and the relative activity of caspase-3 between the treated groups in bladder cancer cells. These findings indicate that ETS-1 may only play vital roles in the migration and invasion of bladder cancer, and did not affect the proliferation and apoptosis.

It should be noted that the results of the correlation of ETS-1 expression with the malignant phenotype of bladder cancer in our study is contrary to the findings from Aysegul S, et al. They reported that most of the low-grade and noninvasive bladder cancer largely expressed ETS-1, and that high-grade and invasive bladder cancer showed a frequent decrease or loss of ETS-1expression [19]. But they only used immunohistochemical staining to investigate the expression of ETS-1 and did not perform the functional experiments in their studies. The expression of ETS-1 was not associated with grade of bladder cancer (*P* = 0.202) (Table 2) in our study. More clinic samples should be needed to reveal the correlation of ETS-1 expression pattern with the clinical-pathological characteristics in bladder cancer.

**Table 2:** Summary of clinical-pathological features of bladder cancer patients

No.	Sex	Age	Grade	Stage	No.	Sex	Age	Grade	Stage
1	M	59	Low	T2bN0M0	22	M	70	Low	TaN0M0
2	F	74	High	T3aN0M0	23	M	68	Low	T2bN0M0
3	M	69	High	T2bN0M0	24	M	57	High	T3aN0M0
4	M	66	High	T2bN0M0	25	F	41	Low	T2aN0M0
5	M	62	High	T4aN0M0	26	M	73	High	T2bN0M0
6	M	71	High	T2bN0M0	27	M	66	High	T3aN2M0
7	M	65	High	T2bN0M0	28	M	72	Low	T1N0M0
8	M	58	High	T4aN3M0	29	F	62	Low	T4N0M0
9	M	57	High	T4aN0M0	30	F	73	High	T2aN0M0
10	M	64	High	T3aN0M0	31	M	74	High	T2bN0M0
11	M	75	High	T2bN0M0	32	M	67	Low	T1N0M0
12	M	58	High	T4aN0M0	33	F	75	High	T1N0M0
13	M	63	High	T3aN0M0	34	M	72	High	T3aN1M0
14	M	63	High	T2bN0M0	35	M	78	High	T2aN0M0
15	M	66	High	T2bN0M0	36	M	53	Low	T1N0M0
16	M	48	Low	T1N0M0	37	F	61	High	T3aN0M0
17	M	53	High	T1N1M0	38	F	81	Low	T1N0M0
18	M	66	High	T1N0M0	39	M	56	High	T4aN0M0
19	M	65	High	T3aN0M0	40	M	49	Low	T1N0M0
20	F	79	High	T4aN0M0	41	M	55	High	T1N0M0
21	F	64	High	T2aN0M0	42	M	65	High	T1N0M0

*No.*, patient number. *M*, male. *F*, female. *Age*, years old. *Grade*, the 2004 World Health Organization classification. *Stage*, American Joint Committee on Cancer TNM classification.

In conclusion, the results of this research demonstrate that ETS-1 is highly expressed in the bladder cancer compared to matched normal bladder tissue, and promotes bladder cancer cell migration and invasion. Hence, ETS-1 should play oncogenic roles in human bladder cancer and it can be used as a therapeutic target for treating human bladder cancer. The future works are still needed to study the molecular mechanisms of ETS-1 which maybe a candidate biomarker for bladder cancer in the clinic.

## MATERIALS AND METHODS

### Patient samples

42 patients with bladder urothelial carcinomas were included in the study. Bladder cancer tissue and paired normal bladder tissue from each patient were snap-frozen in liquid nitrogen instantly after partial or radical cystectomy. Written informed consents were obtained from all the patients. The clinical-pathological characteristics of the patients are summarized in Table 2. The study was approved by the Institutional Review Board of Shenzhen Second People's Hospital.

### Cell lines and culture conditions

Bladder cancer T24, 5637 and UMUC-3 cells were purchased from the Institute of Cell Biology, Chinese Academy of Sciences (Shanghai, China). T24 cell line and UMUC-3 cell line were cultured in Dulbecco's Modified Eagle Medium (Gibco, Grand Island, NY, USA) supplemented with 10% (v/v) fetal bovine serum (Gibco, Grand Island, NY, USA) at 37°C in a 5% CO_2_ atmosphere. 5637 cell line was cultured in RPMI-1640 Medium (Gibco, Grand Island, NY, USA) supplemented with 10% (v/v) fetal bovine serum (Gibco, Grand Island, NY, USA) at 37°C in a 5% CO_2_ atmosphere.

### Construction of ETS-1 shRNA vector and cell transfection

Either the small-hairpin RNA (shRNA) targeting ETS-1 or the negative control shRNA targeting no known sequence were cloned into the pGPU/GFP/Neo vector (GenePharma, Shanghai, China). The ETS-1 shRNA sequence was 5′-CTGATGTAAGGCAATTAAT-3′ [20]. The bladder cells lines were incubated with either ETS-1 shRNA or negative control shRNA using LipoFiter™ Liposomal Transfection Reagent (Hanbio, Shanghai, China) according to the protocol.

### Construction of lentivirus-mediated ETS-1over-expression vector and cell transduction

Human ETS-1 cDNA (NM_001143820) was amplified, purified, and inserted into a lentiviral vector encoding enhanced GFP [21, 22]. The recombinant lentiviral vector expressing ETS-1 (ETS-1 over-expression vector) and the empty vector (Negative control) were constructed from Genechem Co., Ltd (Shanghai, China). The packaging, purification, and titer determination of the recombinant lentiviruses were carried out in HEK293T cells as previously described [23, 24]. Finally, the titers of the recombinant lentiviruses were 2 × 10^8^ and 1 × 10^9^ infectious units/mL, respectively.

Bladder cancer cells were cultured in 6-well plates (2 × 10^5^/well) and infected with the lentivirus at a multiplicity of infection (MOI) of 20 for 24 h. Cells were selected using puromycin (Sigma-Aldrich, St. Louis, MO, USA) for getting stable ETS-1expression. At last, ETS-1 expression in the infected cells was confirmed by means of qRT-PCR.

### Total RNA extraction and reverse transcription

Total RNA was extracted from the tissue samples, the transfected cells and the infected cells by using TRIzol™ reagent (Invitrogen, Carlsbad, CA, USA) according to the manufacturer's instructions. cDNA was converted from total RNA by using the PrimeScript^™^RT reagent Kit with gDNA Eraser (TaKaRa, Otsu, Shiga, Japan) according to the instructions.

### Real-time quantitative polymerase chain reaction (qRT-PCR)

qRT-PCR analyses were conducted with SYBR Premix Ex Taq™II (TaKaRa, Otsu, Shiga, Japan). The primer sequences were as follows: ETS-1 primers [25] forward: 5′-TCATTTCTTTGCTGCTTGGA-3′, reverse: 5′-AAGCCGACTCTCACCATCAT-3′; β-actin primers [26] forward: 5′-GCGAGAAGATGACCCAGAT-3′, reverse: 5′-GAGGCGTACAGGGATAGC-3′. qPCR was performed in a total reaction volume of 20 μl, including 2 μl of First-Strand cDNA, 0.8 μl of forward primer, 0.8 μl of reverse primer, 10 μl of 2 × SYBR Premix Ex Taq™II, 0.4 μl of 50 × ROX Reference Dye II and 6μl of double-distilled water. The reactions were performed in triplicate by using the ABI PRISM 7000 Fluorescent Quantitative PCR System (Applied Biosystems, Foster City, CA, USA). β-actin was chosen as the internal control. The average value in each triplicate was used to calculate the relative amount of ETS-1 using the 2^−ΔΔCt^ methods.

### Cell migration assay

The treated cells were seeded on the 6-well plates (5 × 10^5^/well). At about 90% confluent cells, a clean line was created by using a sterile 200 μl pipette tip at 5 h post transfection. The migration of cells was monitored using a digital camera system and imaged at the time of 0 h and 16 h. The cell migration distance (mm) was calculated using the software program HMIAS-2000. Experiments were performed at least three times.

### Cell invasion assay

About 1 × 10^5^ treated cells with 100 μl serum-free medium were plated into the upper chambers (24-well insert, pore size 8 μm, Corning) which were added with Matrigel (1:8, 50 μl/well, BD Bioscience, San Jose, CA, USA). Concurrently, the lower chambers were filled with 500 μl medium containing 10% fetal bovine serum. Cells were cultured at 37°C in a 5% CO_2_ atmosphere for 48 hours. Cells under the surface of the lower chamber were washed with 1 × PBS, fixed with 4% paraformaldehyde for 20 min, stained with 0.1% crystal violet for 25 min, and then washed 3 times. Invaded cells were observed under the inverted microscope and imaged. Afterwards, each chamber with the invaded cells was soaked into 1 ml 33% acetic acid for 10 min to wash out the crystal violet. 100 μl/well 33% acetic acid were added into 96-well plates, and the absorbance was measured at a wavelength of 570 nm using a microplate reader (Bio-Rad, Hercules, CA, USA). Experiments were performed in triplicate.

### Cell proliferation assay

The effects of down-regulation or up-regulation ETS-1 on cell proliferation were examined by Cell Counting Kit-8 (CCK-8) (Beyotime, Shanghai, China) according to the previous studies [27, 28]. The treated cells were seeded in a 96-well plate (5 × 10^3^/well) and cultured in normal medium. At 0, 24, 48, and 72 h after transfection, 15 μl of CCK-8 was added into each well of 96-well plate and the cells were cultured for 1 hour. Absorbance was measured at a wavelength of 450 nm using a microplate reader (Bio-Rad, Hercules, CA, USA). Assays were repeated at least three times.

Cell proliferation was also tested by 5- ethynyl-2-deoxyuridine (EdU) incorporation assay using the corresponding kit (Ribobio, Guangzhou, China). The EdU incorporation assay was carried out according to the previous studies [29, 30]. Finally, the cells were observed using fluorescence microscopy. All experiments were performed in three times.

### Cell apoptosis assay

The effects of down-regulation or up-regulation ETS-1 on cell apoptosis were detected by calculating the activity of caspase-3 using the Caspase-3 enzyme-linked immunosorbent assay (ELISA) assay kit (Hcusabio, Wuhan, China) according to the manufacturer's instructions. Absorbance was measured by using a microplate reader (Bio-Rad, Hercules, CA, USA). Data were shown as the ratios between the absorbance of experimental groups and those of negative control groups. Each test was carried out at least three times.

### Statistical analyses

All experimental data from three independent experiments were presented as mean ± standard deviation (SD). All statistical data were analyzed by SPSS 19.0 software (SPSS Inc.Chicago, IL, USA). The ETS-1 RNA expression differences between bladder cancer tissue and matched normal tissue were analyzed using paired samples *t*-test. The relationship between ETS-1 level and clinical-pathologic characteristics in 42 patients with bladder cancer were analyzed by Continuous correction chi-square test. The data of CCK-8 assay were analyzed by ANOVA and independent samples *t*-test was used to analyze other data. A *P* value of less than 0.05 was considered to be statistically significant.
